# Meigs’ syndrome with elevated CA-125 and HE-4: a case report and literature review

**DOI:** 10.3389/fmed.2025.1533388

**Published:** 2025-02-24

**Authors:** Jichang Seong, Abdusattorov Ravshan, Sametdinov Narkhodzha, Kurbanova Saida, Alimov Jamshid, Babanov Bahriddin, Sharobidinov Biloliddin

**Affiliations:** ^1^School of Medicine, Central Asian University, Tashkent, Uzbekistan; ^2^Department of Oncology, Akfa Medline University Hospital, Tashkent, Uzbekistan; ^3^Department of General Surgery, Akfa Medline University Hospital, Tashkent, Uzbekistan; ^4^Department of Radiation Oncology, Republican Specialized Scientific and Practical Medical Center of Oncology and Radiology, Tashkent, Uzbekistan; ^5^Department of Cytological and Histological Diagnostics, Ipsum Pathology, Tashkent, Uzbekistan

**Keywords:** Meigs’ syndrome, CA-125, HE-4, fibroma, pleural effusion, ascites

## Abstract

Meigs’ syndrome is a rare gynecological condition characterized by a benign ovarian tumor, ascites, and pleural effusion, all of which resolve spontaneously after tumor removal. While mildly elevated serum CA-125 levels are frequently observed, levels exceeding 1,000 IU/mL are extremely rare, and concurrent elevation of other tumor markers, such as HE-4, may further complicate its diagnosis. We report a case of Meigs’ syndrome in a 41-year-old premenopausal woman. Initial presenting symptoms included severe dyspnea, abdominal distention, anorexia, and weight loss. Subsequent imaging studies revealed a large right ovarian tumor accompanied by massive ascites and pleural effusion. Serum CA-125 and HE-4 levels were markedly elevated (1,200 IU/mL and 82.1 pmol/L, respectively), with a Risk of Ovarian Malignancy Algorithm (ROMA) score of 25.63%, suggesting advanced ovarian malignancy. Neoadjuvant chemotherapy was initiated, but the tumor continued to grow, necessitating internal debulking surgery. Postoperative histopathology revealed a benign ovarian fibroma, confirming the diagnosis of Meigs’ syndrome. Spontaneous resolution of ascites and pleural effusion occurred by the second postoperative day, and the tumor markers normalized within the next six months. The patient remained disease-free at 2-year follow-up. This case underscores the importance of considering Meigs’ syndrome in patients with markedly elevated tumor markers, an ovarian tumor unresponsive to chemotherapy, and concomitant ascites and pleural effusion. Early recognition and surgical intervention are critical for accurate diagnosis and optimal management of this rare condition.

## Introduction

First reported by Joe Vincent Meigs in 1937, Meigs’ syndrome is characterized by the triad of a benign ovarian tumor, ascites, and pleural effusion (1, 2). In the following years, the official diagnostic criteria for Meigs’ syndrome were established as follows: (1) the presence of a benign ovarian tumor, specifically fibroma, thecoma, granulosa cell tumor, or Brenner tumor; (2) concurrent ascites and pleural effusion; and (3) spontaneous resolution of ascites and pleural effusion after tumor removal (1, 3, 4). Pelvic or abdominal tumors other than those recognized in Meigs’ syndrome, when associated with ascites and pleural effusion, are classified as pseudo-Meigs’ syndrome (1, 4). It has been estimated that Meigs’ syndrome arises in approximately 1% of benign ovarian tumors (1, 2). Common presenting symptoms include dyspnea, dry cough, fatigue, poor appetite, weight loss, and painful abdominal distension (1, 4, 5). Due to its rarity, understanding of Meigs’ syndrome mostly relies on case reports, leaving its pathophysiology largely unclear (1, 2, 6, 7).

Meigs’ syndrome presents with several diagnostic challenges. Ascites and pleural effusion in the presence of an ovarian tumor often suggest advanced or disseminated malignancies (8). Cancer antigen (CA)-125, a tumor marker often elevated in epithelial ovarian cancers, is also frequently elevated in Meigs’ syndrome, further complicating its differentiation from ovarian malignancies (1, 9). However, CA-125 levels exceeding 1,000 IU/mL in Meigs’ syndrome are extremely rare (9). A limited number of studies have also documented the elevation of human epididymis protein-4 (HE-4) in Meigs’ syndrome (5, 6). HE-4 is used alongside CA-125 to improve sensitivity in detecting ovarian cancers (10). The concurrent elevation of CA-125 and HE-4, therefore, raises significant suspicion for ovarian malignancies, making its diagnosis even more challenging. In this study, we present a case of Meigs’ syndrome with elevated CA-125 and HE-4 mimicking ovarian malignancy, and we review similar reports to broaden the understanding of Meigs’ syndrome.

## Case presentation

A 41-year-old premenopausal woman presented to the hospital with a 14-month history of severe dyspnea, abdominal distention, loss of appetite, and weight loss. Her obstetric history included regular menarche that started at 13 years of age and three uncomplicated deliveries, following which she opted for an intrauterine device (IUD) for contraception in 2019. She denied any chronic illnesses, including diabetes mellitus, viral hepatitis, and tuberculosis, and had no history of allergies, surgeries, or recent travel. She was a non-smoker and did not consume alcohol or illicit drugs. Her family history was negative for cancer. Physical examination revealed an absent breath sound and dullness to percussion on the right lung, indicating possible pleural effusion. Her abdomen was severely distended with evidence of shifting dullness and a fluid wave, and pelvic examination identified a firm, 14 × 12 cm mass extending to the umbilicus.

**Figure 1:**
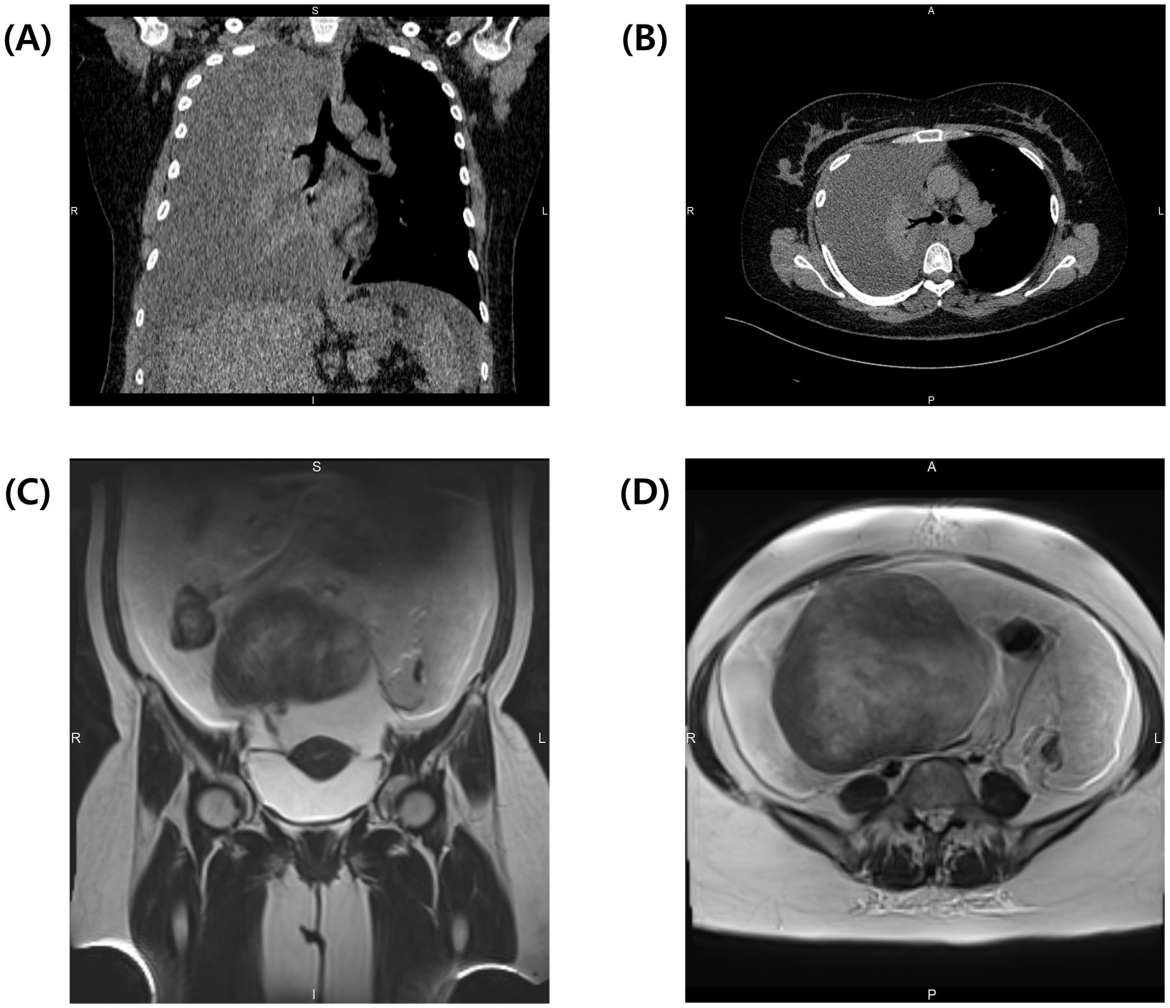
Preoperative CT of the thorax demonstrates complete collapse of the right lung due to massive pleural effusion. **(A)** Coronal view and **(B)** transverse view of the thorax at the level of tracheal bifurcation. Preoperative T2-weighted MRI of the abdomen reveals massive ascites and a solid tumor. **(C)** Coronal view shows the connection between the tumor and the uterus. **(D)** Transverse view of the abdomen shows a large solid tumor measuring 14.5 × 15.5 × 14.3 cm.

Computed tomography (CT) of the chest showed a complete collapse of the right lung due to a significant pleural effusion, while the left lung appeared clear (Figures 1A,B). Immediate thoracentesis drained approximately 2 L of yellowish fluid, of which 100 mL was sent for cytology. Abdominal magnetic resonance imaging (MRI) revealed a solid 14.5 × 15.5 × 14.3 cm mass originating from the right ovary with massive ascites (Figures 1C,D). Paracentesis drained 8 L of ascitic fluid, of which 100 mL was sent for cytological analysis. Given the high suspicion of ovarian malignancy with possible metastasis, serum tumor markers were analyzed: carcinoembryonic antigen (CEA) was 1.07 ng/mL (reference range 0–5.09 ng/mL), CA 19–9 was 4.57 IU/mL (reference range 0–41 IU/mL), CA-125 was markedly elevated at 1,200 IU/mL (reference range 0–35 IU/mL), and HE-4 was 82.1 pmol/L (reference range 0–70 pmol/L). The Risk of Ovarian Malignancy Algorithm (ROMA) score was 25.63%, suggesting a high likelihood of ovarian malignancy. Cytology from the pleural effusion showed numerous reactive mesothelial cells, neutrophils, and lymphocytes, while the ascitic fluid revealed several atypical cells, raising concerns for pleural and peritoneal metastasis.

**Figure 2:**
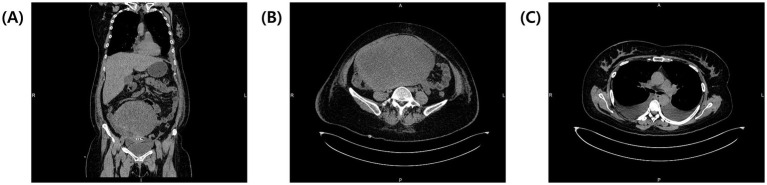
Post-NACT CT of the thorax and abdomen. **(A)** Coronal view showing the enlarged tumor compressing surrounding structures. **(B)** Transverse view of the abdomen with enlarged tumor now measuring 20.5 × 13.5 × 17.3 cm. **(C)** Transverse view of the thorax showing bilateral pleural effusions at the level of tracheal bifurcation.

**Figure 3:**
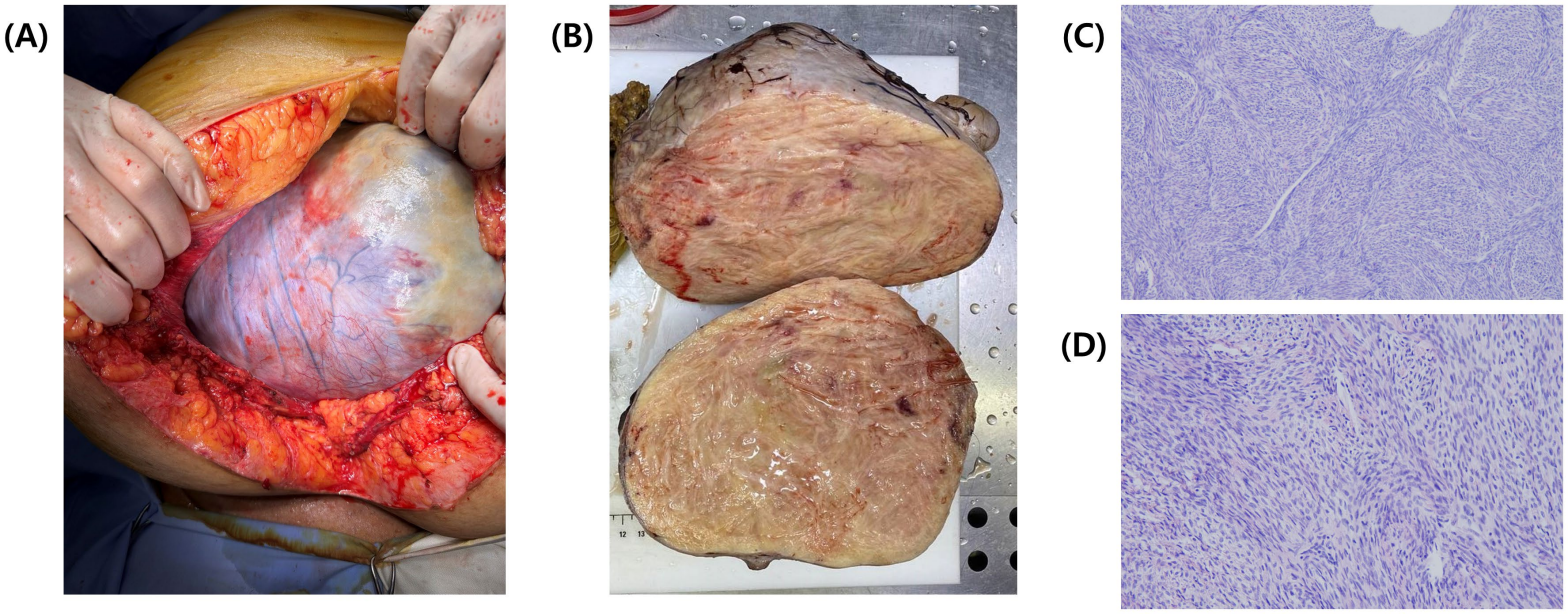
Intraoperative and postoperative images of the tumor. **(A)** Intraoperative view of the tumor during open laparotomy. **(B)** Postoperative cross-section of the tumor showing a solid, densely packed yellowish mass. **(C)** Hematoxylin and eosin (H&E) stained histological section of the resected tumor, viewed under light microscopy at 20x magnification and **(D)** at 40x magnification.

Exploratory laparoscopy was initially planned, but persistent pleural effusion, ascites, and the patient’s worsening condition rendered surgery unfeasible. According to the National Comprehensive Cancer Network (NCCN) guidelines, neoadjuvant chemotherapy (NACT) was initiated with paclitaxel (175 mg/m^2^) and carboplatin (AUC 5) to reduce the tumor burden and control effusions. During two cycles of NACT, the patient developed a pulmonary embolism, which was managed with fraxiparine and rivaroxaban. Unfortunately, NACT proved ineffective; the ovarian mass increased to 20.5 × 13.5 × 17.3 cm, compressing surrounding structures (Figures 2A,B), and pleural effusions extended to left hemithorax (approximately 800 mL), necessitating bilateral thoracentesis (Figure 2C). Given the disease progression despite NACT, internal debulking surgery (IDS) was pursued despite the high operative risk. Intraoperative findings revealed a solid tumor that originated from the right ovary (Figure 3A). The procedure included bilateral salpingo-oophorectomy, total abdominal hysterectomy, omentectomy, and parietal biopsy. The surgery was uneventful, and postoperative histopathology confirmed a benign right ovarian fibroma with no evidence of malignancy (Figures 3B,C). The final diagnosis of Meigs’ syndrome was made.

**Figure 4:**
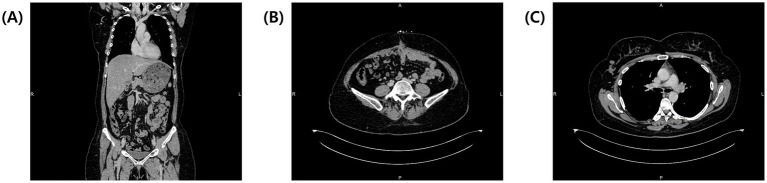
Contrast-enhanced CT at 6 months follow-up. **(A)** Coronal view of the entire body showing complete resolution of pleural effusion and ascites. **(B)** Transverse view of the abdomen showing no tumor recurrence and complete ascites resolution. **(C)** Transverse view of the thorax at the tracheal bifurcation level showing full resolution of pleural effusions.

The patient’s pleural effusion and ascites resolved spontaneously by the second postoperative day. She was discharged on the seventh postoperative day, and follow-up at six months showed normalized tumor markers (CA-125 at 16.6 IU/mL and HE-4 at 30.7 pmol/L), with no evidence of recurrence or residual effusion on contrast enhanced-CT (Figures 4A–C). She remained disease-free at a 2-year follow-up.

## Review of the literature

We identified a total of 49 cases of Meigs’ syndrome with elevated CA-125 levels from 45 reports published between 2015 and 2024, sourced from PubMed (6, 7, 9, 11–32) and Google Scholar (33–52). A summary of these cases is provided in Supplementary Table 1. The ages of the patients ranged from 11 to 85 years. The most common ovarian tumor histopathology was fibroma (25 cases), followed by fibrothecoma (18 cases), granulosa cell tumor (four cases), and thecoma (two cases). Many reports did not specify the weight of the tumor, although one case described a tumor weighing as much as 36 kg (27). Tumors were most commonly located in the right adnexa (27 cases), followed by the left adnexa (16 cases), with four cases involving bilateral adnexal tumors (20, 23, 26, 35). CA-125 levels ranged from 57 IU/mL to 4,900 IU/mL, with 11 cases having CA-125 levels exceeding 1,000 IU/mL.

Pleural effusion was most often seen in the right hemithorax (26 cases), followed by bilateral hemithorax (17 cases). Two cases presented with isolated left pleural effusion (40, 43). Ascitic fluid volumes ranged from 300 mL to 20,000 mL. The majority of patients underwent unilateral or bilateral salpingo-oophorectomy along with hysterectomy. Postoperative discharge occurred mostly within one week, with the shortest discharge time being two days (20, 23) and the longest being 33 days (19).

Postoperative complications included pulmonary embolism and deep vein thrombosis (30), as well as one death due to sepsis and multiorgan failure (26). No patient died directly from Meigs’ syndrome. No cases required additional postoperative treatment except one patient who received monthly goserelin and bevacizumab for one year postoperatively (31). Pleural effusion and ascites resolved spontaneously within days to weeks after surgery, and the CA-125 levels returned to the reference range within weeks to months. Patients were followed for several months to years, with no recurrence noted in the longest follow-up period of 10 years (20).

## Discussion

First described by Joe Vincent Meigs in 1937, Meigs’ syndrome is defined as the presence of a benign ovarian tumor, ascites, and pleural effusion, all of which resolve spontaneously after tumor resection (1). Historically, only fibroma, thecoma, granulosa cell tumor, and Brenner tumor were recognized as diagnostic criteria for Meigs’ syndrome (1). However, other benign ovarian tumors, such as hemangioma (53), sclerosing stromal tumor (54), and Sertoli-Leydig tumor (55), have been reported as Meigs’ syndrome. Due to the inconsistent application of terminology and the usage of different clinicopathological presentations to define the same entity, Krenke et al. proposed a new classification of Meigs’ and related syndromes. This classification includes Demons-Meigs’ syndrome, pseudo-Meigs’ syndrome, and incomplete Meigs’ syndrome, based on precise case definitions (4).

Previous studies and our review of the literature suggest that fibroma, including cellular fibroma, is the most frequently associated tumor in the Meigs’ syndrome with elevated CA-125 levels. Abad et al. reviewed 14 cases from 11 reports published between 1989 and 1997, identifying seven cases of fibroma (including cellular fibroma), three cases of fibrothecoma, three cases of thecoma (including luteinized thecoma), and one case of granulosa cell tumor (56). Similarly, Moran-Mendoza et al. analyzed 27 cases from 17 reports published between 1989 and 2004, identifying 18 cases of fibroma (including cellular fibroma), four cases of thecoma (including luteinized thecoma), three cases of fibrothecoma, and one case each for granulosa cell tumor and Brenner tumor (57). Benjapibal et al. reviewed 28 cases from 18 reports published between 1989 and 2007, identifying 19 cases of fibroma (including cellular fibroma), four cases of thecoma (including luteinized thecoma), three cases of fibrothecoma, and one case each for granulosa cell tumor and Brenner tumor (58). Cha et al. analyzed 43 cases from 33 reports published between 1989 and 2012, identifying 22 cases of fibroma (including cellular fibroma), seven cases of fibrothecoma, four cases of thecoma (including luteinized thecoma), three cases of granulosa cell tumor (including juvenile form), two cases of Brenner tumor, and five cases of sclerosing stromal tumor, the latter not meeting the traditional Meigs’ syndrome criteria (59). Although overlapping cases may affect these trends, our literature review from 2015 onward further supports that fibroma is the most commonly associated tumor in Meigs’ syndrome.

Meigs’ syndrome is often accompanied by elevated CA-125 levels, making it difficult to distinguish from advanced ovarian malignancies. However, levels exceeding 1,000 IU/mL are exceptionally rare (23, 57, 60). CA-125 is an antigenic tumor maker commonly expressed in epithelial ovarian malignancies (61). In addition, it is widely expressed in other coelomic epithelia, including the fallopian tubes, peritoneum, pleural, pericardium, colon, kidney, and stomach (61, 62). This broad expression reduces its sensitivity and limits its utility to the detection of early ovarian cancer in postmenopausal women (62). The exact mechanism of CA-125 elevation in Meigs’ syndrome remains unclear, yet a study done by Liou et al. proposed that this elevation is likely due to the mesothelial expression rather than tumor expression, as immunohistochemical staining of the tumor specimens for CA-125 was negative (63). Moreover, studies have explored the correlation between CA-125 levels and disease presentation, but the findings remain controversial. Liu et al. demonstrated that higher levels of CA-125 were positively correlated with the volume of ascites (63). Similarly, Shang et al. reported a significant correlation between serum CA-125 level and the volume of ascites (5). In contrast, Iavarone et al. did not find any correlation between CA-125 levels and any major elements in Meigs’ syndrome (9). Kortekaas et al. also found no correlation between CA-125 levels and ascites; however, the authors suggested a potential link between CA-125 levels and hydrothorax (17). Although our patient presented with massive ascites, pleural effusion, and a markedly high CA-125 level, our literature review did not reveal a correlation between CA-125 levels and the volume of either pleural effusion or ascites.

Several hypotheses have been proposed to explain the formation of ascites and pleural effusion in Meigs’ syndrome. Meigs et al. suggested that ascites form through fluid leakage from the edematous fibroma or tumoral obstruction of intraperitoneal lymphatic drainage (64, 65). Subsequently, pleural effusion develops as the ascitic fluid passes into the pleural cavity through the right-side predominant congenital diaphragmatic defects or through the lymphatic pathways (64). A study demonstrated that vasoactive factors, such as vascular endothelial growth factor (VEGF) and fibroblast growth factor (FGF), along with the inflammatory cytokine interleukin-6 (IL-6), were significantly elevated in ascitic fluid compared to serum and pleural fluid, suggesting that tumor-secreted vasoactive factors increase peritoneal vascular permeability and lead to transudation and ascites formation (66). Moreover, these vasoactive factors were found less in pleural fluid, supporting the hypothesis that pleural effusion arises from the secondary passage of ascitic fluid through the diaphragm (66). A study also demonstrated that pleural effusion predominantly occurs on the right side, with the majority of cases being exudative (4). Consistent with previous findings, our literature review revealed that pleural effusion predominantly affects the right hemithorax. In our patient, the nature of the fluid was not determined since fluid cytology revealed atypical cells, and the clinical presentation strongly indicated advanced ovarian malignancy. Studies to date have focused on the nature and location of pleural effusion but have not explored its association with disease progression. In our patient, an initial right-sided pleural effusion progressed to bilateral effusion as the tumor size increased. This observation suggests a potential link between effusion dynamics and tumor growth. Therefore, we carefully propose that the location of pleural effusion may reflect disease progression.

According to the NCCN clinical practice guidelines for ovarian cancer, NACT may be considered for patients who are not good candidates for surgery (67). For instance, NACT is used in patients with poor performance status to reduce tumor burden, improve overall condition, and lower perioperative risk (67). Specifically, NACT with IDS is the primary treatment for patients with advanced-stage malignancy, as well as for those unsuitable for primary debulking surgery due to advanced age, frailty, poor performance status, or comorbidities (67, 68). Considering our patient’s poor performance status, we initiated NACT; however, the tumor continued to grow, rendering the NACT treatment ineffective. We found only one similar case in the literature. Moran-Mendoza et al. diagnosed a patient with advanced epithelial ovarian cancer based on a significantly elevated CA-125 level (1,808 U/mL) and initiated induction chemotherapy due to the patient’s unsuitability for surgery. Despite administering three cycles of paclitaxel and carboplatin, six cycles of vinorelbine, and two cycles of gemcitabine, there was no reduction in the tumor mass, although CA-125 levels significantly declined (57). The authors advised that while false positives are rare in patients with malignant ascites cytology and elevated CA-125 level, chemotherapy should not be initiated unless a definitive cancer diagnosis is made. They also emphasized that even with a high suspicion of ovarian malignancy, a minimally invasive biopsy is crucial when the benign disease is clinically suspected (57). We support these recommendations and further suggest that if a tumor mass fails to respond early during chemotherapy, Meigs’ syndrome should be initially considered, prompting an earlier transition to surgical tumor resection rather than completing multiple cycles of ineffective chemotherapy. A timely shift to surgery in such cases may improve patient outcomes and avoid unnecessary treatment delays.

HE-4 has emerged as a promising biomarker for early detection of ovarian malignancy (69). Studies have reported that HE-4 is minimally expressed in the epithelial tissues of the respiratory and reproductive organs but is significantly overexpressed in ovarian tumors, offering greater reliability than CA-125 (10). Thus, the combination of HE-4 and CA-125 is widely recognized for its improved sensitivity in detecting ovarian malignancies, with elevated levels of both markers strongly suggesting ovarian cancer (10, 70). However, elevated HE-4 levels can also be observed in Meigs’ syndrome. A retrospective study of nine patients with Meigs’ syndrome found that HE-4 levels were significantly higher than those in the ovarian fibrothecoma group but notably lower than those in the ovarian cancer group (5). Surprisingly, two studies have documented the concurrent elevation of both HE-4 and CA-125 levels in Meigs’ syndrome. Danilos et al. reported a HE-4 level of 116.3 pmol/L and a CA-125 level of 2,310 IU/mL (6), while Slaoui et al. reported a HE-4 level of 122.1 pmol/L and a CA-125 level of 1,028 IU/mL (49). To the best of our knowledge, this is the third reported case of concurrent HE-4 and CA-125 elevation in Meigs’ syndrome. Although only three cases are available, including ours, all studies showed CA-125 levels exceeding 1,000 IU/mL, suggesting a possible correlation between CA-125 levels above 1,000 IU/mL and elevated HE-4 levels. Furthermore, our case demonstrates that while elevated HE-4 and CA-125 levels are highly indicative of ovarian malignancy and are extremely rare in Meigs’ syndrome, they may still occur. Hence, despite elevated tumor markers suggesting ovarian malignancy, clinicians must always consider Meigs’ syndrome in the differential diagnosis when an ovarian tumor is accompanied by ascites and pleural effusion to avoid misdiagnosis and unnecessary radical surgery.

Tumor biomarkers play a crucial role in cancer screening, early diagnosis, prognosis assessment, recurrence detection, and monitoring treatment effectiveness (71). Identifying a comprehensive biomarker pool and expanding its clinical utility may pave the way for more effective therapeutic strategies and personalized treatment approaches (72). For instance, new targeted therapies, such as PARP inhibitors, have proven highly effective in destroying ovarian cancer cells and have become an essential part of maintenance treatment (72, 73). To date, no specific biomarkers exist for the accurate preoperative diagnosis of ovarian fibroma or fibrothecoma (74). Therefore, future research on identifying ovarian fibroma biomarkers may improve the accurate diagnosis of Meigs’ syndrome, reducing misdiagnosis and guiding appropriate clinical management.

In conclusion, our findings suggest a potential relationship between the location of pleural effusion and disease progression and suggest that elevated CA-125 levels above 1,000 IU/mL may coincide with elevated HE-4 levels in Meigs’ syndrome. Regardless of tumor marker levels, Meigs’ syndrome should always be considered in the differential diagnosis when an ovarian tumor is present with concurrent ascites and pleural effusion. Furthermore, if the tumor fails to respond to NACT initiated under the suspicion of advanced ovarian malignancy, Meigs’ syndrome should be considered early in the treatment process, prompting a timely shift toward surgical resection.

## Data Availability

The original contributions presented in the study are included in the article/Supplementary material, and further inquiries can be directed to the corresponding author.

## Supplementary material

SUPPLEMENTARY TABLE 1 Summary of the clinical characteristics of 49 patients diagnosed with Meigs’ syndrome with elevated CA-125 levels.
